# Long-Term Safety and Efficacy of CT-Guided I^125^ Radioactive Seed Implantation as a Salvage Therapy for Recurrent Head and Neck Squamous Carcinoma: A Multicenter Retrospective Study

**DOI:** 10.3389/fonc.2021.645077

**Published:** 2021-07-01

**Authors:** Yuliang Jiang, Peng Zhen, Jinchao Dai, Yixing Li, Shifeng Liu, Junma Xu, Yufeng Wang, Suqing Tian, Yue Cui, Zhe Ji, Fuxin Guo, Bin Qiu, Haitao Sun, Jinghong Fan, Junjie Wang

**Affiliations:** ^1^ Department of Radiation Oncology, Peking University 3^rd^Hospital, Beijing, China; ^2^ Department of Radiation Oncology, Chifeng Cancer Hospital, Chifeng, China; ^3^ Department of Nuclear Medicine, Qingdao Central Hospital, Qingdao, China; ^4^ Department of Nuclear Medicine, Yichang First People’s Hospital, Yichang, China; ^5^ Department of Interventional Radiology, Affiliated Hospital of Qingdao University, Qingdao, China; ^6^ Department of Respiratory, Jintan District People’s Hospital, Changzhou, China; ^7^ Department of Nuclear Medicine, Xuzhou Cancer Hospital, Xuzhou, China

**Keywords:** recurrent head and neck cancer, brachytherapy, radioactive seed implantation, squamous carcinoma, radiotherapy

## Abstract

**Purpose:**

To investigate the safety and efficacy of CT-guided I^125^ radioactive seed implantation (RSI) as a salvage therapy for recurrent head and neck squamous carcinoma (rHNSC) after external beam radiotherapy (EBRT) or surgery.

**Materials and Methods:**

This is a multicenter retrospective study of 113 patients (83 males; median age 57 years) with rHNSC who underwent CT-guided I^125^ RSI between February 2003 and December 2017. Of the included patients, 107 patients previously received EBRT and 65 patients received surgery and all were ineligible or rejected for salvage surgery and/or repeat EBRT.

**Results:**

During a median follow-up duration of 20 months (range, 3-152 months), 87 patients died. The 1-, 2-, 3-, and 5-year local control rate were 57.4%, 41.8%, 29.3%, and 15.2%, respectively. The median time to progression was 15 months [95% confidence interval (CI), 6.1-23.9 months]. The median overall survival (OS) was 20 months (95% CI, 12.4-27.6 months). The 1-, 2-, 3-, and 5-year OS rate were 63.6%, 44.6%, 29.9%, and 21.7%, respectively. Univariate and multivariate analyses revealed that KPS score and postoperative D90 were significantly associated with patients’ OS. The complications were mainly grade I/II skin and mucosal reactions: 18 cases (15.9%) of grade I/II and eight cases (7.0%) of grade III radiation dermatitis, and 14 cases (12.4%) of grade I/II and three cases (2.7%) grade III mucosal reactions. No grade IV or severer complications were found.

**Conclusion:**

CT-guided I^125^ RSI may be safe as a salvage therapy for rHNSC after EBRT/surgery, yielding promising efficacy compared with historical data. KPS score and postoperative D90 may be significantly associated with OS.

## Introduction

Head and neck squamous carcinoma (HNSC) is the sixth most common cancer and accounts for over 600,000 new cancer cases and 350,000 deaths worldwide each year (1, 2). Despite the high local control rate of HNSC, recurrent HNSC (rHNSC) still occurs in 20%-35% of the patients after surgery/chemo-radiotherapy (3). Though long-term survival is becoming more common in HNSC, the outcome for rHNSC is still very poor (4). Salvage therapy for recurrent disease may preferentially benefit this subset; unfortunately, treatment options are limited (3).

Salvage surgery leads to a substantial improvement in outcomes for rHNSC although it only can be used in highly selected patients (5). In the context of radiation used for patients with rHNSC, the ideal technique should be able to deliver therapeutic doses to the targets with doses as low as possible to the organs at risk to improve the prognosis of rHNSC (6). Re-irradiation using external beam radiotherapy (EBRT) is promising though still challenging for managing lesions raised in previously irradiated fields. Therefore, EBRT can only be considered in well-selected patients but at the high cost of toxicity (3, 7).

Brachytherapy offers dosimetric advantages with very sharp radiation dose gradients compared with conventional external-beam techniques (8). Given the introduction of a brachytherapy-treatment planning system (BT-TPS), optimal dose distribution can be realized using radioactive seed implantation (RSI) for various cancers. Notably, I^125^ radioactive seed implantation (RSI) becomes an optimal tool for prostate carcinoma (9). Therefore, I^125^ RSI may be used as a salvage treatment for rHNSC due to its focused irradiation on the tumor and rapid dose fall-off at distance from the sources, limiting dose exposure to surrounding tissues (8). Several single-institute retrospective studies were previously published for patients with rHNSC (10–13). Here, the multicenter retrospective study reported a long-term outcome of CT-guided I^125^ RSI for patients with rHNSC.

## Materials and Methods

### Patients Selection Criteria

This is a retrospective study of 113 patients [83 males; median age 57 years (range, 26-83 years)] with rHNSC treated with CT-guided I^125^ RSI in six centers in China between February 2003 and December 2017 (a major center contributes 84 patients and the remaining centers contribute 1-20 cases each). Of the included patients, 107 patients previously received EBRT and 65 patients received surgery. The patient’s characteristics were listed in **Table 1**. The local control rate, time to progression, overall survival (OS) rate, and complications were analyzed. The evaluation of tumor response was based on the Response Evaluation Criteria in Solid Tumors (RECIST) v1.1 (14). Local control was defined as complete response, partial response, and stable disease. Time to progression was defined as the time from the RSI procedure until tumor progression. Overall survival was defined as the time from the RSI procedure until the time of death from any cause or last follow-up. Complications were determined by the Common Terminology Criteria for Adverse Events (CTCAE) v4.0 (CTCA) (15).

**Table 1:** Clinical characteristics of the 113 patients.

	n	%
Gender		
Male	83	73.5
Female	30	26.5
Median age (years)	57 (26-83)	
Median KPS	80 (60-90)	
Primary tumor		
Nasopharyngeal carcinoma	31	27.4
Oral carcinoma	27	23.9
Hypopharyngeal carcinoma	16	14.2
Laryngeal carcinoma	15	13.3
Nasal/pararnasal sinuses carcinoma	10	8.8
Lymph-node metastasis of unknown	7	6.2
Skin carcinoma	3	2.7
salivary carcinoma	2	1.8
Oropharyngeal carcinoma	1	0.9
Thyroid cancer	1	0.9
Pathology		
Squamous cell carcinoma	111	98.2
Adenosquamous carcinoma	2	1.8
Previous surgery	65	57.5
Once	50	
Twice and/or more	15	
Previous radiotherapy	103	91.2
Once	86	
Twice and/or more	17	
Previous chemotherapy ± target therapy	62	54.8
Median previous cumulative dose (Gy)	66 (30-160)	

The indication for I^125^ RSI in this study was all follows: (1) Aged 18-85 year-old with KPS≥70; (2) Pathological/radiological recurrent tumor in patients with pathological diagnosed HNSC after prior EBRT/surgery, with the lesion diameter less than 7cm; (3) CT revealed a possible needles pathway capable for RSI; (4) Patients without metastasis or with stable metastasis (less than 3 lesions); (5) Expected survival time more than 3 months; and (6) Ineligibility or rejection of salvage surgery and/or repeat EBRT (Ineligibility commonly referred to uncertain R0 resection or tumor invasion of large blood vessels or poor conditions after discussion by a Multidisciplinary Team minimum consisting of a surgeon, oncologist, and radiologist). All patients had signed an informed consent form for RSI, which stated the advantages and disadvantages of RSI. Contraindication was one of the following: (1) Severe organ dysfunction, (2) Coagulation dysfunction, (3) Active infection, (4) Mental illness, or (5) Extensive necrosis/ulcer formation/skin rupture. The study was approved by the ethics committee of each center and the requirement to obtain written informed consent to the participant of the study was waived.

### Pre-Operative Preparation and Pre-Plan

All patients received a blood routine test, coagulation function, and biochemistry examination before RSI to rule out contraindication. The patients were immobilized on a CT simulator couch with a custom vacuum lock bag in the supine or lateral position. Both plain and contrast CT scans were performed with 5mm thickness before 1-2 days of RSI. The image data were transmitted into BT-TPS (Beijing University of Aeronautics and Astronautics and Beijing Astro Technology Co., Ltd) for pre-plan according to planning system source data originated from the official recommendation of the American Association of Physicists in Medicine (AAPM) (16, 17). The clinical tumor volume (CTV) was formed by expanding 5-6 mm with three dimensions from gross tumor volume (GTV). The doses received by 90% of GTV (GTV D_90_) were supposed to be as close to the prescription dose as possible whereas the doses received by OARs were kept as low as possible. The median prescription dose was 120Gy (range, 110-160 Gy) and the activity of I^125^ seed (size 4.5mm×0.8mm and half-life time 59.4 days; Beijing Atom high Tech Pharmaceutical Company Inc., China) was 0.22-0.83 mCi (median 0.65mCi).

### CT-Guided I^125^ RSI

The RSI protocol was as follows: (1) patients were set up on the CT-simulator and immobilized with a vacuum pad. Local anesthesia was carried out and 2-3 reference needles were inserted 2-3 cm into the patient’s body per pre-plan; (2) CT scan was performed to determine the accurate position of the reference needles; (3) When the reference needles’ position was mismatched with pre-plan, the adjustment in real-time was made until the deviation less than 2 mm; (4) The seed needles were all inserted into the targets; (5) CT scan was performed again to confirm the position of all needle tips, similarly, if the deviation was more than 2 mm, an adjustment would be made; (6) ^125^I seed was delivered with applicator (Mick 200-TPV Applicator, Mick Radio-Nuclear Inc., US) in a receding manner; and (7) CT scan was performed again to confirm the distribution of I^125^ seeds in the targets. The CT images were transferred into the BT-TPS for post-plan doses’ evaluation. Patients would be discharged 1-2 days after RSI. All procedures followed the recommendations by the International Commission on Radiological Protection (18). The dosimetric parameters, e.g. D90, were recognized.

### Follow-Up

Patients were routinely followed with CT/MRI in 3-month intervals for the first 2 years, 6-month intervals from 3 to 5 years, and then followed up annually after RSI. The evaluation of tumor response was based on the images obtained at 3 months after RSI.

### Statistical Analysis

A Chi-square test was used for the analysis of tumor response. Survival was estimated using the Kaplan-Meier method and univariate analysis was conducted using the log-rank test, and multifactor analysis was conducted using Cox regression. P<0.05 was considered significant.

## Results

Of the 113 patients included, 87 patients died and 26 survived (20 patients were lost to follow-up since August 2019 and were counted as truncated numbers) during a median follow-up duration of 20 months (range, 3-152 months).

### Tumor Response

**Figure 1:**
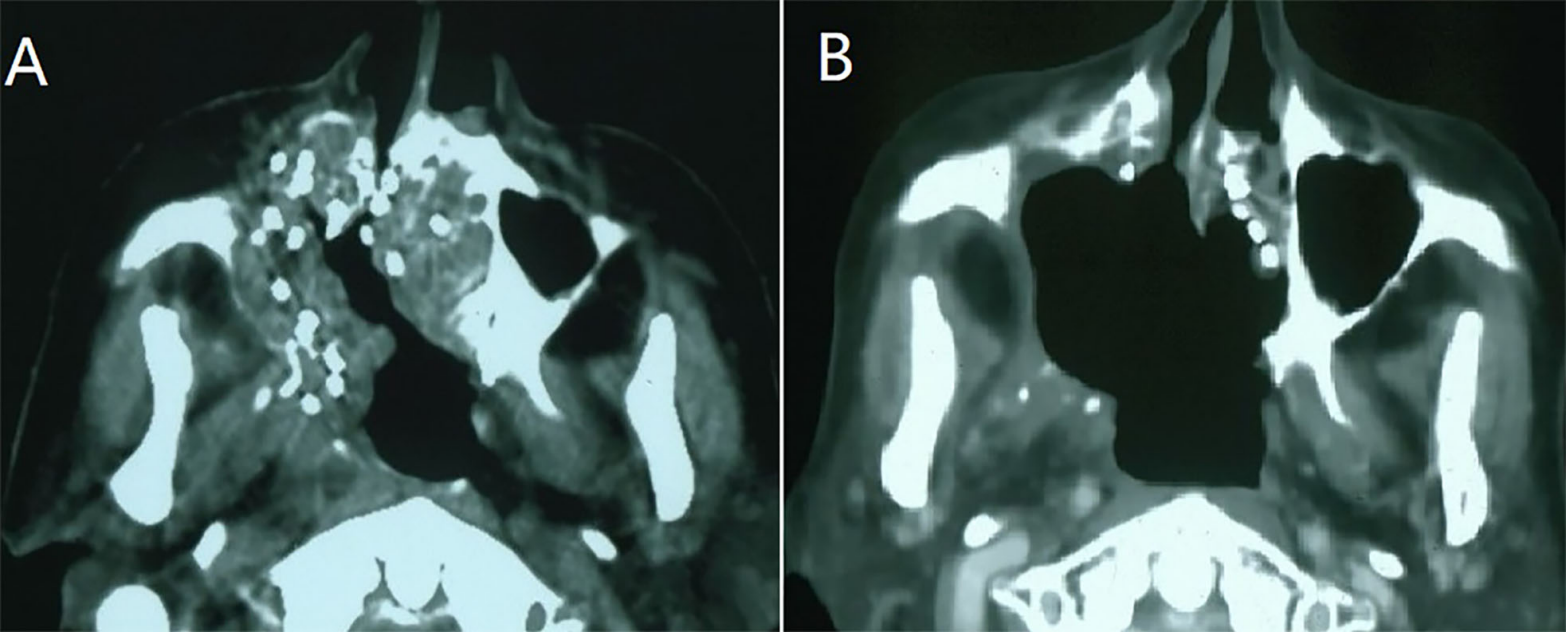
A case of recurrent maxillary sinus squamous carcinoma following surgery and adjuvant radiotherapy. **(A)** CT-guided I^125^ seeds were implanted as a salvage treatment; **(B)**: Three months after the seed implantation, partial response was observed.

**Figure 2:**
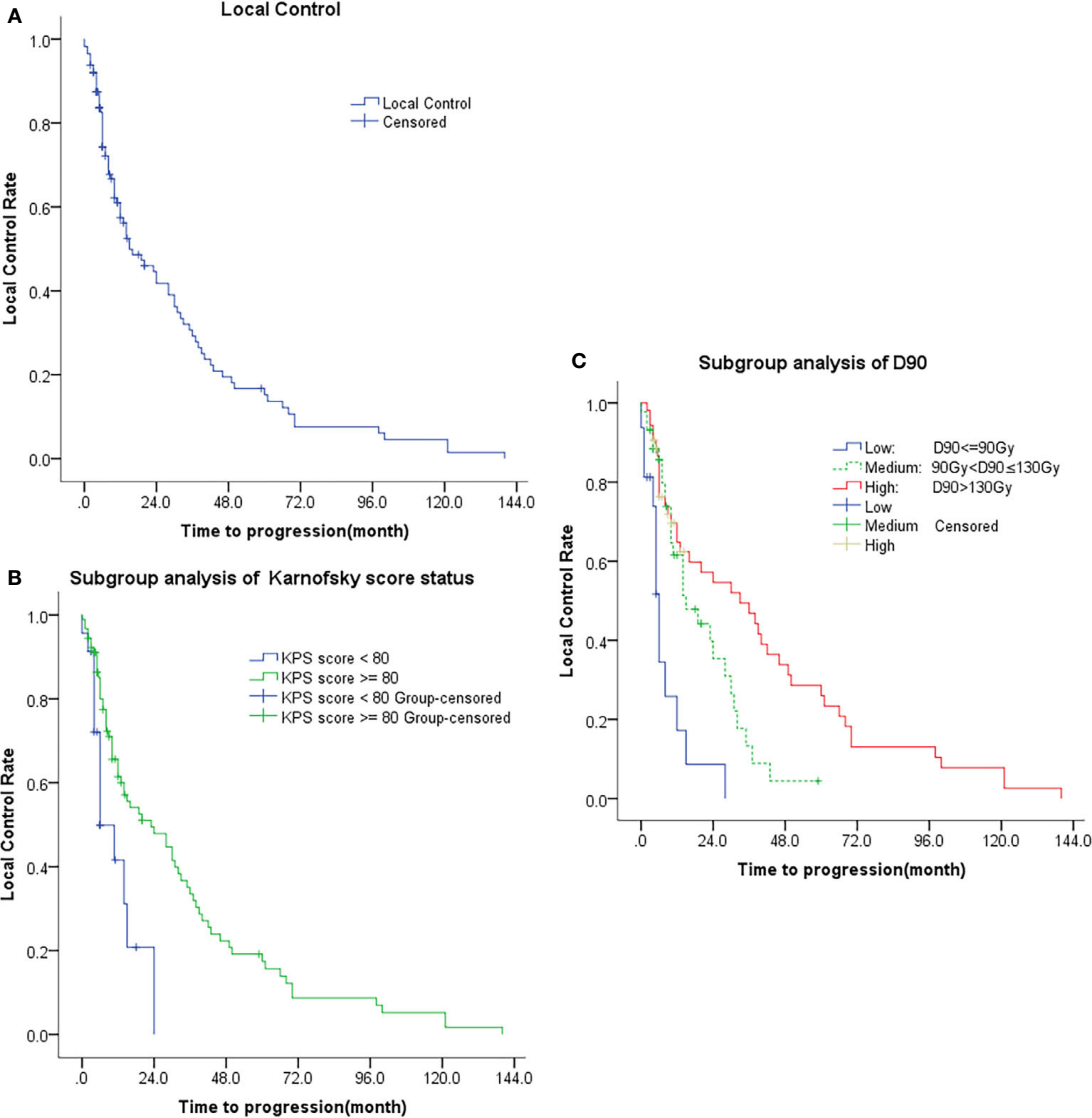
Kaplan-Meier plots of time to progression; **(A)** Analysis of all patients; **(B)** Subgroup analysis of KPS (KPS < 80 and KPS ≥ 80); **(C)** Subgroup analysis of D90 (D90 ≤ 90Gy, 90Gy < D90 ≤ 130Gy, and D90 > 130Gy).

There were 20 cases of complete response, 74 cases of partial response, 13 cases of stable disease, and six cases of progressive disease, **Figure 1**. The 1-, 2-, 3-, and 5-year local control rate were 57.4%, 41.8%, 29.3%, and 15.2%, respectively. The median time to progression was 15 months [95% confidence interval (CI), 6.1-23.9 months], **Table 2**. Univariate analysis showed that age, sex, previous surgery, radiotherapy, chemotherapy, and site of recurrence were not associated with local control (p = 0.311, 0.079, 0.582, 0.511, 0.697, and 0.738, respectively). The local control of patients with D90 > 90Gy was significantly better than that of patients with D90 ≤ 90Gy (p=0.000). The local control rate of the group with good performance (KPS score ≥ 80) was better than that of the group with poor performance (KPS score < 80) (p = 0.003), **Table 3** and **Figure 2**.

**Table 2:** Clinical outcomes of the 113 patients.

	n	%
Tumor response		
CR	20	17.7
PR	74	65.5
SD	13	11.5
PD	6	5.3
Local control rate		
1-year	-	57.4%
2-year	-	41.8%
3-year	-	29.3%
5-year	-	15.2%
Time to progression	Median 15 months	-
Overall survival	Median 20 months	-
Overall survival rate		
1-year	-	63.6%
2-year	-	44.6%
3-year	-	29.9%
5-year	-	21.7%
Complications		
Skin		
Grade I, II	18	15.9
Grade ≥III	8	7.0
Mucosal		
Grade I, II	14	12.4
Grade ≥III	3	2.7
Others		
Grade I, II	0	-
Grade ≥III	0	-

CR, complete response; PR, partial response; PD, progression disease; SD, stable disease.

**Table 3:** Subgroup analysis of tumor response.

Factors	Group	n	Local control rate (%)				p
			1-y	2-y	3-y	5-y	
Age (years old)	<60	66	53.7	38.5	24.9	11.3	0.311
	≥60	47	62.4	46.3	35.6	20.8	
KPS	<80	23	41.6	20.8	–	–	0.003
	≥80	90	61.4	47.9	33.5	17.4	
Implantation site	Primary tumor	69	55.3	40.9	30.1	12.5	0.738
	Lymph nodes	44	60.8	43.6	27.7	15.8	
D90 (Gy)	≤90	16	17.2	–	–	–	0.000
	>90, ≤130	44	61.5	35.3	13.3	–	
	>130	53	64.8	54.6	46.8	26.0	
Previous surgery	Yes	65	65.1	48.2	30.7	13.1	0.582
	No	48	46.9	32.8	24.6	15.4	
Previous radiotherapy	Yes	103	56.6	43.7	30.6	15.9	0.511
	No	10	58.0	–	–	–	
Previous chemotherapy/target therapy	Yes	62	55.7	43.1	30.4	15.2	0.697
	No	51	58.9	40.6	28.1	15.6	

### Survival

**Figure 3:**
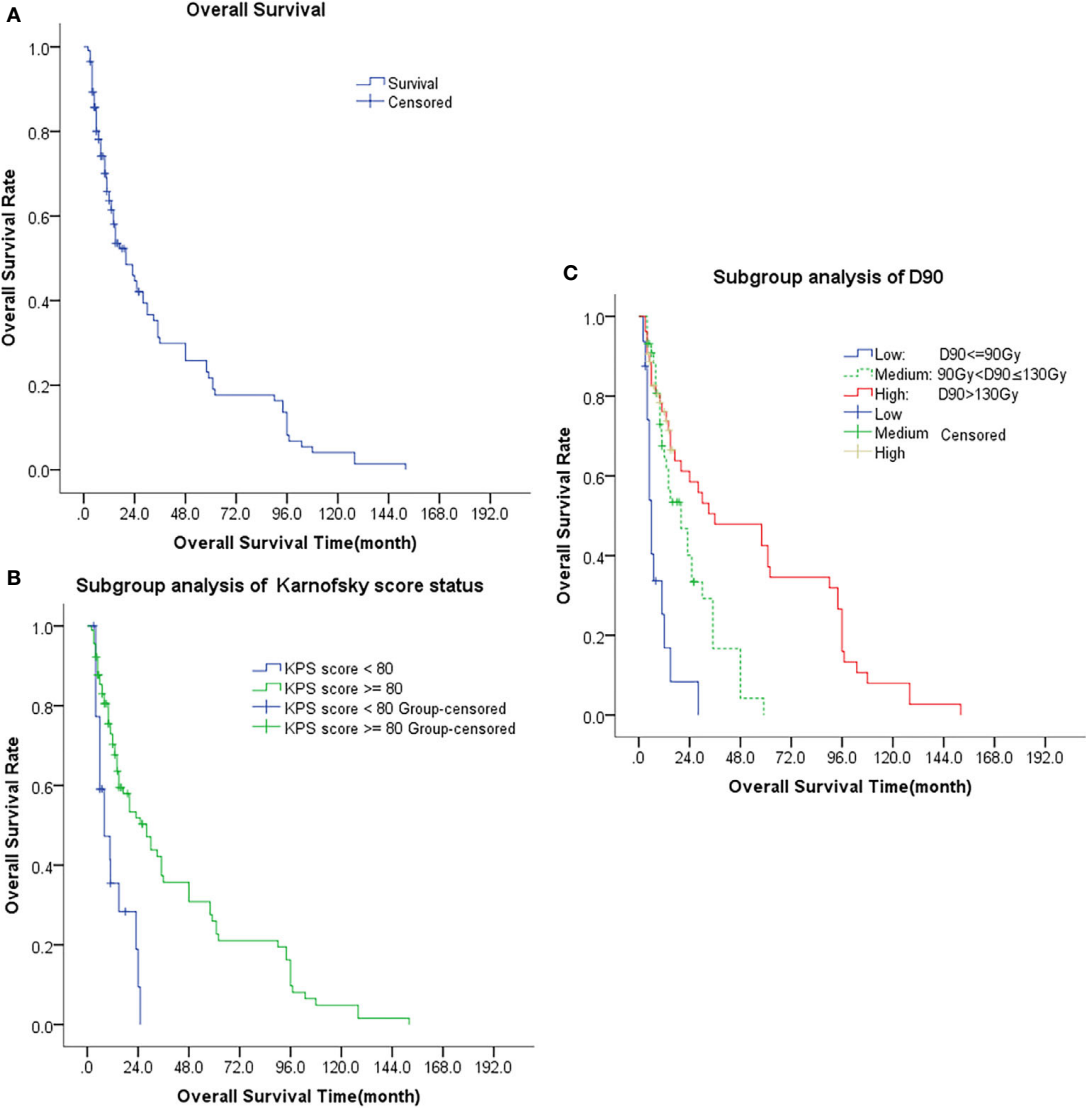
Kaplan-Meier plots of overall survival curves; **(A)** Analysis of all patients; **(B)** Subgroup analysis of KPS (KPS < 80 and KPS ≥ 80); **(C)** Subgroup analysis of D90 (D90 ≤ 90Gy, 90Gy < D90 ≤ 130Gy, and D90 > 130Gy).

The median OS was 20 months (95% CI, 12.4-27.6 months). The 1-, 2-, 3-, and 5-year OS rate were 63.6%, 44.6%, 29.9%, and 21.7%, respectively, **Table 2**. Univariate analysis showed that age, sex, previous surgery/radiotherapy/chemotherapy, and site of recurrence were not associated with OS (p = 0.422, 0.793, 0.994, 0.328, 0.614, and 0.708, respectively). The OS of patients with D90 > 90Gy was significantly better than that of patients with D90 ≤ 90Gy (p<0.001) and the OS of patients with good performance (KPS score ≥80) was also better than that of patients with poor performance (KPS score < 80) (p<0.001). Multivariate analyses also revealed that only KPS score and postoperative D90 were significantly associated with patients’ OS (both p<0.001), **Table 4** and **Figure 3**.

**Table 4:** Subgroup analysis of overall survival.

Factor	Group	n	Survival rate (%)				p
			1-y	2-y	3-y	5-y	
KPS	<80	23	35.5	9.5	–	–	0.000
	≥80	90	70.3	51.8	35.7	26.0	
D90 (Gy)	≤90	16	16.8	8.4	–	–	0.000
	>90, ≤130	44	64.7	40.1	16.7	–	
	>130	53	76.1	58.5	47.8	42.5	

### Complications

Local pain at the implantation site was found in five cases (6.0%) during RSI. During follow-up, the complications were mainly grade I/II skin and mucosal reactions: 18 cases (15.9%) of grade I/II and eight cases (7.0%) of grade III radiation dermatitis, and 14 cases (12.4%) of grade I/II and three cases (2.7%) of grade III mucosal reactions. No grade IV or severer complication were found, **Table 2**.

## Discussion

The multicenter retrospective study reported a favorable long-term result of CT-guided I^125^ RSI as a salvage therapy for rHNSC. During a median follow-up duration of 20 months, patients experienced a relatively high rate of local control rate and OS rate. KPS score and postoperative D90 were significantly associated with local control and patients’ OS. The complications were mainly minor skin and mucosal reactions.

With the development of modern radiation techniques, such as 3-dimensional conformal radiotherapy (3D-CRT) or intensify modulated radiotherapy (IMRT), reirradiation is applied for rHNSC after EBRT. Reirradiation using EBRT with or without chemotherapy improved the local control rates in half of the patients and long-term survival in selected patients (19, 20). EBRT as a salvage option is promising for rHNSC in the previously irradiated area although it still poses a great challenge as it is hard to deliver sufficient doses to the targets while sparing the normal tissues, especially for tumors previously treated with full doses. The treatment-related toxicity was as high as 20-30% (20, 21). I^125^ RSI may have the following advantages: (1) Radioactive seeds once implanted were continuously irradiating the tumors without interval compared with EBRT; (2) The radiation dose of the target boosted high enough to achieve an ablative effect while sparing the normal tissue; and (3) The therapy was minimally invasive and resulted in a short hospital stay for the patients. However, a direct comparison was not available.

In the largest multi-institutional phase II trial of Radiation Therapy Oncology Group for unresectable rHNSC managed with repeat EBRT (reirradiation) and chemotherapy (RTOG-9610) (22), the 2-year and 5-year OS were only 15.2% and 3.8%. The worst acute toxicity was grade 4 in 17.7% and grade 5 in 7.6%. Grade 3 and 4 late toxicities were found in 19.4% and 3.0%, respectively. In a randomized phase III trial (GORTEC 98-03) comparing repeat EBRT (reirradiation) plus chemotherapy *versus* methotrexate in 57 patients with rHNSC (23), 1-year OS was only 23% *versus* 22%. Sixteen patients (28%) experienced clinical grade ≧̸3 late toxicities. This was a retrospective study of 327 patients with recurrent and metastatic (RM) HNSC (24). All patients received at least one line of active treatment (eg, surgery, concurrent chemoradiotherapy, or radiotherapy/chemotherapy alone/with surgery) and those receiving only the best supportive care were excluded. The median OS was only 14 months with a favorable group (46%) and 10 months with an unfavorable group (54%), which seems inferior to that reported here (i.e. 20 months). Compared with these historical data of irradiation, the current study reveals CT-guided I^125^ RSI may be promising as a salvage therapy for rHNSC after EBRT/surgery.

Previous evidence of RSI for rHNSC were all single-institute retrospective studies. The largest study by Ji et al. (10) reported 101 patients treated with CT-guided I^125^ RSI for recurrent head and neck cancer (rHNC) after EBRT. The median previous cumulative external radiation dose was 66 Gy, and the median D_90_ after RSI was 117 Gy. The 5-year local control rate was 26.6%, the median survival time was 15 months, and the 5-year OS rate was 15.5%. The 5- year local control rate was 11.5% (2-year) when D_90_ < 120 Gy and was 44.2% when D_90_ = 120 Gy (p=0.001). There were 26 (25.7%) cases of skin/mucosa ulceration among them; 15.8% were grade I to II, 7.9% were grade III, and only 2% were grade IV. Fourteen cases suffered from pain (13.9%) and two cases with dry mouth (2%). The report was the only published study for I^125^ RSI with over 100 cases and the outcomes were similar to the present study. This was a study by Jiang et al. (13). involving 64 patients, with 81 rHNC in total, treated with permanent I^125^ RSI under ultrasound guidance. The median follow-up period was 14 months. The total response rate was 80% (27% complete response and 53% partial remission), which is similar to that of our study. The 1-, 3-, and 5-year tumor control rates were 75.2%, 73.0%, and 69.1%, respectively, which is better than that reported here; this may be attributed to the difference of pathological pattern and recurrent sites between the two studies. This is because the results for cervical lymph node recurrence were better than those for primary rHNC reported in her study, with 5-year local control rates of 72.7% and 39.9%, respectively. The 1-, 3-, and 5-year overall survival rates were 57.4%, 31%, and 26.6%, respectively, with a median survival of 20 months. D90 was also found to be an independent prognostic factor of the efficacy. Grade I/II skin reactions were seen in 11 patients (17%) who had received external beam radiotherapy before. Other severe complications were absent. These survival and complication outcomes were all similar to the present study, while Grade IV skin ulceration was seen in two patients in their study. In the present study, the rate and severity of toxicity were relatively low and minor. The potential reasons may include: (1) RSI was conducted with a 0.5-1 cm safety margin away from the organ at risk under CT-guidance; (2) The activity of I^125^ seed was kept as 0.4-0.5 mCI when the tumor invaded the organ at risk; (3) Patients with tumor-infiltrated skin or mucosa were excluded, so skin or mucosa ulceration may be avoided; and (4) The indication for I^125^ RSI was strict with an appropriate diameter of the tumor or needle pathway. Within the above screening criteria, CT-guided RSI may be a feasible and safe treatment for patients with rHNSC after EBRT; further study is warranted.

There are several limitations to this study. Firstly, as a multicenter study, the majority (74%, 84/113) of the patients were included from a major center, which may result in potential bias. Secondly, the study is a retrospective study with a single arm and 20 patients were lost to follow-up since August 2019, which may also lead to a certain bias. Thirdly, RSI is used as salvage therapy for the patients, so there was no control group with the current standard treatment of salvage surgery or repeat EBRT. However, the study was the first multicenter study with over 100 cases investigating the long-term safety and efficacy of RSI for rHNSC obtaining favorable outcomes, therefore, prospective studies with a control group are needed in the future.

## Conclusion

CT-guided I^125^ RSI may be safe as a salvage therapy for rHNSC after EBRT/surgery, yielding promising efficacy compared with historical data. KPS score and postoperative D90 may be significantly associated with patients’ outcomes.

## Data Availability Statement

The raw data supporting the conclusions of this article will be made available by the authors, without undue reservation.

## Ethics Statement

The studies involving human participants were reviewed and approved by Peking university Third Hospital Medical Science Rearch Ethics Committee. The ethics committee waived the requirement of written informed consent for participation.

## Author Contributions

YJ, PZ, and JW conceived and designed the study. YJ, PZ, JD, YL, SL, JX, YW, ST, YC, ZJ, FG, BQ, HS, JF, and JW performed the study and data collection. YJ is responsible for statistical analysis. YJ, BQ, and JW wrote the paper. JW reviewed and edited the manuscript. All authors contributed to the article and approved the submitted version.

## Funding

National Key Research and Development Plan of China (Grant No. 2019YFB1311300) to JW supports the implementation (e.g., labor cost and data collection) and publication of the project.

## Conflict of Interest

The authors declare that the research was conducted in the absence of any commercial or financial relationships that could be construed as a potential conflict of interest.
